# Schizophrenia: A Review of Social Risk Factors That Affect Women

**DOI:** 10.3390/bs13070581

**Published:** 2023-07-12

**Authors:** Alexandre González-Rodríguez, Mentxu Natividad, Mary V. Seeman, Jennipher Paola Paolini, Ariadna Balagué, Eloïsa Román, Eduard Izquierdo, Anabel Pérez, Anna Vallet, Mireia Salvador, José Antonio Monreal

**Affiliations:** 1 Department of Mental Health, Mutua Terrassa University Hospital, Fundació Docència I Recerca Mutua Terrassa, University of Barcelona (UB), 5 Dr. Robert Square, 08221 Terrassa, Spain; mnatividad@mutuaterrassa.cat (M.N.); jpaolini@mutuaterrassa.cat (J.P.P.); abalague@mutuaterrassa.es (A.B.); eloisaroman@mutuaterrassa.cat (E.R.); eizquierdo@mutuaterrassa.cat (E.I.); aperez@mutuaterrassa.cat (A.P.); avallet@mutuaterrassa.cat (A.V.); msalvador@mutuaterrassa.cat (M.S.); jamonreal@mutuaterrassa.cat (J.A.M.); 2 Centro de Investigación Biomédica en Red de Salud Mental (CIBERSAM), 08221 Terrassa, Spain; 3 Department of Psychiatry, University of Toronto, 605 260 Heath Street West, Toronto, ON M5P 3L6, Canada; 4 Institut de Neurociències, Universitat Autònoma de Barcelona (UAB), 08221 Terrassa, Spain

**Keywords:** schizophrenia, psychosis, social determinants, women

## Abstract

Social risk factors are long-term or repeated environmental exposures in childhood and youth that change the brain and may, via epigenetic effects, change gene expression. They thus have the power to initiate or aggravate mental disorders. Because these effects can be mediated via hormonal or immune/inflammatory pathways that differ between men and women, their influence is often sex-specific. The goal of this narrative review is to explore the literature on social risk factors as they affect women with schizophrenia. We searched the PubMed and Scopus databases from 2000 to May 2023 using terms referring to the various social determinants of health in conjunction with “women” and with “schizophrenia”. A total of 57 studies fulfilled the inclusion criteria. In the domains of childhood and adult abuse or trauma, victimization, stigma, housing, and socioeconomics, women with schizophrenia showed greater probability than their male peers of suffering negative consequences. Interventions targeting appropriate housing, income support, social and parenting support, protection from abuse, violence, and mothering-directed stigma have, to different degrees, yielded success in reducing stress levels and alleviating the many burdens of schizophrenia in women.

## 1. Introduction

Social risk factors relevant to the development of chronic psychotic disorders such as schizophrenia have begun, after a period of relative neglect, to receive serious attention in the psychiatric literature [1,2,3,4]. These social determinants of health have been identified as combinations of parental maltreatment or neglect, conflict and exposure to violence, stressful life events, emigration and acculturation, negative interpersonal interactions, lack of social support and social capital, educational and employment discrimination, adverse socioeconomic conditions, urbanicity, poor neighborhood and housing, and demographic factors (age, gender, minority status) [2,4,5]. Some risks, but not all, may be preventable through protective social programming, education, and training [6,7,8].

It should be noted that psychosocial risk factors affect health through biological networks while, at the same time, biology (e.g., gene expression) can determine environmental outcomes such as the quality of interpersonal relations and the probability of achieving satisfactory social status [9,10,11]. Because both nature and nurture differ between men and women, the effects of social determinants of schizophrenia may hypothetically also differ in men and women; this is the literature that we explore in this review.

## 2. Materials and Methods

### 2.1. Search Strategy

We conducted a narrative review using the PubMed and Scopus databases from January 2000 to May 2023. PubMed captures most of the medical literature whereas Scopus links the scholarly literature across a wide variety of related disciplines. Combining these two databases provided access to the vast majority of evidence in the field of psychosocial risk factors for schizophrenia. 

The following search terms were used: Abuse OR Adoption OR Bullying OR Discrimination OR Employment OR Immigration OR Neglect “Social Capital” OR “Social Defeat” OR “Social Network” OR “Socioeconomic Status” OR Stigma OR Urbanicity AND Schizophrenia AND Women. The search strategy was conducted by A.G.R and M.N. In order to capture current thought and practice, we only included studies published from the year 2000 onwards. 

### 2.2. Inclusion and Exclusion Criteria

Papers were included if they addressed or referred to the influence of social determinants of schizophrenia in women. Publications (editorials, experimental studies, reviews, and theoretical articles) in four languages, namely English, Spanish, German, and French, were included. Exclusions were papers published in other languages or published prior to 2000, regardless of their relevance to our topic.

### 2.3. Data Collection and Extraction

**Figure 1:**
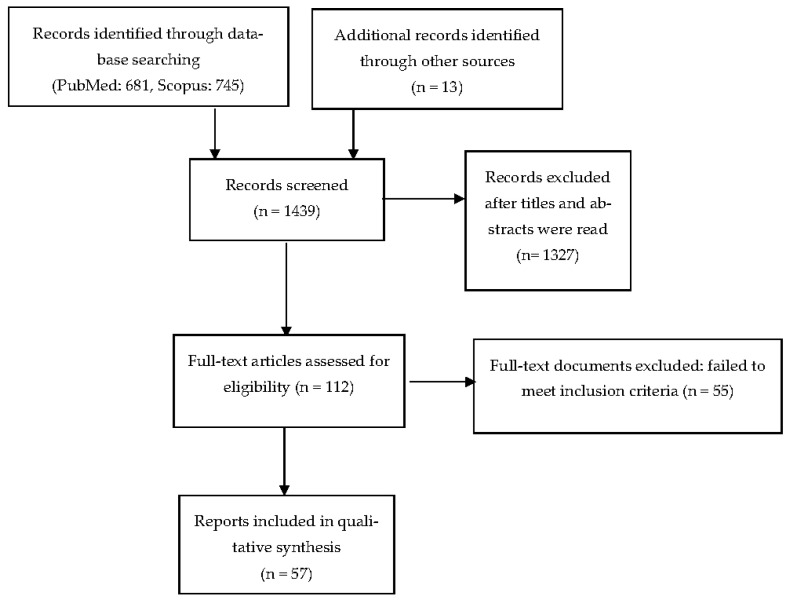
Flow diagram of included studies.

The PubMed and Scopus databases were first searched for relevant titles and abstracts and were manually screened by two authors (A.G.R and M.N.). We next screened full-text documents and searched reference lists in the hope of finding additional eligible studies. Disagreements as to relevance or eligibility were resolved by team consensus. The screening and selection process is shown in Figure 1.

## 3. Results

A total of 1439 records were screened through PubMed (*n* = 681), Scopus (*n* = 745), and reference lists (*n* = 13). Fifty-seven studies were ultimately included.

Table 1 illustrates the role of social determinants of schizophrenia from a gender perspective. Table 2 presents the effects of trauma/abuse on women diagnosed with schizophrenia.

### 3.1. The Role of Social Capital and Social Defeat in Schizophrenia

Social capital is an umbrella term that describes the quality, density, and diversity of social networks, membership in a cohesive group, and sense of belonging, as well as confidence in ready assistance in times of trouble or need [12]. Eliacin and collaborators conducted a qualitative study in London, U.K., in a sample of 62 African-Caribbean women with schizophrenia. Audiotaped interviews probed their personal view of their social capital [13]. What emerged from their responses was an account of general fragmentation in communal living (economic deprivation, high rates of crime, violent riots, racism, police brutality, single parenthood, failed expectations). The authors’ conclusion was that community fragmentation led to feelings of social defeat, a concept taken from animal studies and defined in humans as the hurtful experience of being continually excluded from membership in the majority social group [14]. Social defeat has been positively associated with the prevalence of schizophrenia in both men and women [14]; it is not uniquely associated with schizophrenia since social connectedness is important to the much wider construct of general health [15].

While the sex-specific response to social defeat has not been explored in humans, there are numerous publications on sex-specific models of social defeat in rodents. When an aggressive mouse is introduced into a communal cage, the other mice show social avoidance, females more so than males [16]. In humans, it has been shown that men and women are susceptible to different forms of social defeat [17]. Women, in general, are more stressed by relationship failures while men are more affected by status failures. An interesting socio-anthropological perspective on social defeat in women with schizophrenia is presented in Luhrmann [18]. 

### 3.2. The Role of Employment/Unemployment

The loss of employment or failure to gain employment has major financial implications for men and women and leads to major stress. It has never been shown that this can lead to schizophrenia, but it is known that a majority of individuals with schizophrenia of working age are unemployed and that the minority who have steady jobs enjoy a relatively superior (objective) quality of life. This probably reflects the influence of illness severity on employment rather than the effect of work on the quality of life [19]. Holm et al. [20] found that, three years prior to diagnosis, 24% of individuals with schizophrenia were employed but the employment rate dropped after diagnosis, and, after 5 years, only 10% held jobs outside the home. Rates of employment vary because they depend on the precise definition of employment/unemployment. In general, workplace-related issues that shed light on low rates of employment in this population remain underexplored [21]. A qualitative study from Mexico suggests that employment status is influenced by gendered role expectations and that schizophrenia women, for whom working outside the home is not a community expectation, have an easier time than men in coping with unemployment [22].

A qualitative study from India describes the shame and stigmatization that men with schizophrenia express at being unemployed. Women also experienced stigma related to their illness, but it had more to do with marriage prospects and parenting ability [23].

In 2001, Marcotte and Wilcox-Gök found that 5–6 million workers in the U.S. between the ages of 16 and 54 lose, fail to seek, or cannot find employment as a consequence of mental illness [24]. The literature uncovered a further aspect of employment. A study from Malaysia found that more men than women with a schizophrenia diagnosis had jobs, but that the subjective quality of life of men who were employed was significantly *lower* than that of the unemployed [25]. This speaks to the nature of jobs available to men as distinct from those available to women and varies with country, culture, urban versus rural residence, and also with changing economic times. It also importantly speaks to the status of jobs offered to individuals with disabilities such as schizophrenia. Such jobs are often poorly paid and are sometimes less remunerative than disability pensions; they are less secure, more demeaning, and more physically taxing than jobs offered to those without disabilities, and they incur disrespect from employers and co-workers that negatively impacts subjective quality of life. 

Depending on place and time, there may be more available jobs for women with schizophrenia than for their male peers. In a recent paper about male and female veterans with schizophrenia in the U.S., female gender predicted higher occupational functioning, and this was irrespective of baseline symptom severity [26]. 

### 3.3. The Role of Socioeconomic Status

One’s financial means exert multiple influences on health, but this is a complex domain where effective intervention is elusive. In some regions of the world, poverty is a particularly knotty problem for women that profoundly affects their access to prevention and treatment of disease [27]. Several studies have shown the correlation among social inequities and mental health [28,29] as moderated by age, educational status, ethnicity, and physical health. Although clinicians are rarely in a position to improve their patients’ financial means directly, strong advocacy can induce governments to assist with employment, disability credits, affordable accommodation, health insurance coverage, and transportation services for individuals with serious mental illness [30]. 

With respect to male/female differences in the impact of parental financial means, Parrot and Lewine [31] evaluated 84 men and 36 women with schizophrenia. The Watt’s Amherst Modification of the Hollingshead–Redlich Two-Factor Index of Social Position was used to assess parental socioeconomic status. A high socioeconomic status was associated with lesser symptom severity in women but, paradoxically, with increased symptoms, especially negative symptoms, and decreased global function in men. The finding that negative symptoms increased among sons of male high-socioeconomic-status parents illustrates the “paradox of advantage” perspective, which, according to the authors, means that the increased severity of apathy and emotional withdrawal reflects the hopelessness of sons who are unable to meet what they perceive as unreachable parental vocational expectations [32]. The authors also suggest that stigma is induced by situations, such as male unemployment, that society deems to be gender-incongruent. 

### 3.4. The Role of Housing and Homelessness

Social isolation is known to increase schizophrenia risk. The neighborhoods one lives in, the people with whom one lives, how often one moves, and the quality of housing are all important in this respect [33]. Housing is a particularly significant social determinant of mental health for women living with children [34] because the negative effects of poverty, inadequate schooling, isolation from supportive social networks, and harms from environmental hazards can all be passed down from one generation to the next [35]. 

A study from Spain concludes that elements of housing that result in the greatest adverse effect on general health are noise, leaks, and harmful temperatures. Survey respondents also complain about pollution, neighborhood crime, and population density [36]. 

A systematic review of evidence on housing and mental health was published in 2019 [37]. It did not specifically refer to male/female differences, but an earlier study by the same Australian team [38] suggests that the adverse effects of suboptimal housing are more evident in men than in women. 

With respect to homelessness, Tinland and collaborators carried out two multi-center randomized controlled trials of two hundred and eighteen homeless men and women diagnosed with schizophrenia and recruited from four urban centers in France [39,40]. The women with schizophrenia moved more frequently than the men, often shuttling from living with family to living with friends and subsequently returning to family. The women were more likely to suffer from recent physical or sexual assaults, experienced more depression and posttraumatic symptoms, and presented a higher suicide risk and worse physical health than the men [39,40].

Opler et al. [41] also investigated gender differences in schizophrenia among homeless and housed study participants and found less substance abuse, fewer negative symptoms, and older age at first hospitalization in women. These differences may help women to adjust better than men to housing instability. It appears that the negative effects of inadequate housing differ in men and women from different study cohorts.

### 3.5. The Role of Social Networks

Social isolation and feelings of belonging depend on much more than how and where and with whom one lives. Many factors contribute to social community integration, an important predictor of mental health recovery in the context of psychosis.

Using data from the Survey of High Impact Psychosis, an Australian population survey of adults with psychotic disorders, Galletly et al. [42] compared social functioning in the age group between 18 and 49 (*n* = 1478) with that in the 50 to 65 age group (*n* = 347). The older group, which contained a higher proportion of women, had a smaller social network, attributable perhaps to unemployment (no work colleagues), and high divorce rates. Compared to the general population, small social networks were a feature of both men and women with psychosis.

A study from Korea found that the frequency of social contact in a group of approximately 200 patients with mental disorders (50% men and 50% women) was significantly higher in women than in men. Diagnostic categories were not specified in this study [43]. An Egyptian study of community integration among psychiatric patients [44] found a significant relationship with gender, with female patients showing higher levels of community integration, and also recovery, than male patients. The investigators attribute this to a later age of onset in women, theoretically giving female patients a longer opportunity during which to mature, choose a career, build relationships, and become integrated into their community prior to illness onset. Although the authors do not supply a precise diagnosis for their study participants, the comment about onset age suggests psychosis. They cite two studies [45,46] whose findings contradict their own, showing no gender difference in the community integration of psychiatric patients but, here again, there are no precise diagnoses. Until research better defines what and who is being investigated, the issue of gender difference in social networks and community integration remains unclear.

### 3.6. The Role of Stigma and Discrimination

Stigma, negative biases, and social, as well as institutional, discrimination negatively impact schizophrenia severity, but their causes differ in males and females. Men with schizophrenia are ostracized because of the public’s fear of unprovoked verbal threats or physical violence, while women with the same diagnosis are stigmatized by the widespread societal belief that schizophrenia precludes adequate mothering. Such widespread prejudice towards women with schizophrenia undermines sexual relationships, family planning, marriage, pregnancy, labor and delivery, and childcare [47]. The rate of forced separation of children from mothers with schizophrenia is very high in some regions of the world [48], often rationalized by the supposed implications of the diagnosis and not by evidence of neglectful or abusive parenting.

Farrelly et al. and collaborators [49] in London, U.K., carried out a cross-sectional study investigating the association of expected and experienced discrimination in persons diagnosed with schizophrenia as compared to other severe mental illnesses. The Discrimination and Stigma Scale (DISC), the Internalized Stigma on Mental Illness (ISMI) scale, and the Questionnaire on Anticipated Discrimination (QUAD) were used to measure experiences of anticipated, perceived, and objectively defined discrimination. Around 93% of the sample endorsed anticipated discrimination, and 87% reported experiencing discrimination in the year preceding the study. Statistically significant associations were found between the experience of discrimination and its anticipation. Women were more likely than men to do the anticipating. 

A study from India concludes that both men and women with schizophrenia experience stigma but that men are mainly exposed to it in the context of their work roles while women perceive it most in their marital life, during pregnancy and childbirth [23]. An interesting cultural slant is that it is the norm for postpartum women in India to display quasi psychotic behavior (called “sanni” or “janni”) and that such behavior is, therefore, not stigmatized. When it comes to internalized stigma (internalized or self-stigma is when persons with mental illness absorb negative social stereotypic attitudes and apply them to themselves), West et al. [50] found that a larger percentage of women than men (41.4% vs. 34.8%) scored high, but the difference was, in fact, not statistically significant. 

### 3.7. The Role of Emigration, Immigration, and Acculturation

In Norway, Iversen et al., 2003 [51], compared admission rate, length of hospital stays, and diagnosis among immigrants, asylum seekers, and Norwegian-born patients admitted to a psychiatric hospital during the years 1995–2000. Data were obtained from hospital records. A total of 94 immigrants (38 women and 56 men), 39 asylum seekers (10 women and 29 men), and 2920 Norwegians (1698 women and 1222 men) were admitted during that period. The analyses of the diagnoses within the three groups revealed that schizophrenia was an especially common diagnosis in the women from the immigrant group, highlighting migration as a potential risk factor for schizophrenia in immigrant women. 

Most immigration studies, however, suggest that immigration is more difficult for men than for women, perhaps because the male bread-winner role is difficult to maintain in an alien environment. In a recent review, Pence et al. [52] concluded that immigration was more closely associated with psychosis risk in men than in women. The authors warn that this finding should be taken with caution because of the limited number of studies examining this phenomenon, the methodological limitations of the studies, and the lack of consistency across the results. They recommend further investigations.

### 3.8. The Role of Urbanicity

Living in urban vs. rural areas is strongly associated with schizophrenia. Luo et al. [53] analyzed the association between the risk of schizophrenia and urbanicity from the Second National Sample Survey on Disability (SNSSD), a register of prevalence, disability cause, socioeconomic status, health services, and living conditions. The investigators used a standardized formula to quantify and compare various degrees of urbanicity. The mortality rates of schizophrenia decreased with each degree of urbanicity, but they decreased more so in men than in women. Taking marriage rates as an outcome measure, Yang et al. [54] conducted a large epidemiological study of individuals with schizophrenia living in urban (defined as towns or cities) and rural areas of China. Marital status was divided into married, remarried, never married, separated/divorced, and widowed. Urban women were more likely to be unmarried than women from rural areas. An earlier onset of disease in the urban women may have partially accounted for this. 

Pillay and Sargent [55] carried out a retrospective study of one thousand two hundred and thirty-two patients attending one of five mental health clinics in rural South Africa. Fifty-six per cent (*n* = 692) were diagnosed with schizophrenia, with 295 being women (42.6%). The finding of a high rate of schizophrenia in these rural areas was explained by their disadvantaged socioeconomic status. This suggests that socioeconomics may overshadow the effect of urbanicity.

**Table 1:** Summary of the main findings on the association between social risk factors, schizophrenia, and gender.

Social Risk Factors	Influence on Mental Health	Male/Female Influence on Schizophrenia	Study Design	Refs
Social capital and social defeat	Membership in a cohesive group positively influences mental health	Men react with defeat to loss of status; woman are more likely to react to relationship loss	Prospective quality study	[13]
Employment/unemployment	Employment improves objective quality of life and mental health	Women cope better with unemployment than men, and jobs for women may be easier to find	Nationwide population registry, cross-sectional (qualitative and quantitative)	[20,21,22,23,24,25,26]
Socioeconomic status	Low income correlates with low quality of life and health	Higher parental SES correlates with decreased symptom severity in women, but not always in men	Cross-sectional (part of larger projects)	[31,32]
Housing	Homelessness and neighborhood disadvantage correlates with vulnerability to violence, marginalization, and exploitation	Homelessness in women correlates with sexual victimization. Women’s and men’s housing needs differ	Multi-center studies, cross-sectional	[39,40,41]
Social network	Individuals with SMI are frequently single, living alone, and unemployed	Schizophrenia is associated with small social networks, more so in men than in women	Population survey (self-report), cross-sectional	[42,43,44,45,46]
Discrimination	Perceived “otherness” and discrimination lead to isolation and social marginalization	Reasons for discrimination differ in women and men with schizophrenia	Cross-sectional	[23,49,50]
Immigration	Processes of emigration, immigration, and acculturation are major stressors	New immigration is especially difficult for men	Register study	[51,52]
Urbanicity	The higher the degree of urbanicity, the higher the risk for schizophrenia	Urbanicity and socioeconomic disadvantage are risks for men and women	National survey study, population-based study	[53,54,55]

### 3.9. The Role of Abuse/Trauma

#### 3.9.1. Prevalence and Incidence of Childhood Adversities in Women with Schizophrenia

Prokopez and collaborators from Argentina [56] conducted a cross-sectional study of 100 patients with schizophrenia from three health facilities as well as 50 controls. The Adverse Childhood Experiences scale was used to explore the history of physical, emotional, and/or sexual abuse, the history of physical and/or emotional neglect, and the history of household dysfunction. Adverse events in childhood were almost double those found in the control group. Multiple adverse events were associated with persistent auditory hallucinations and fewer negative symptoms in patients of both sexes. Notably, a higher frequency of death ideation and a higher number of suicide attempts were reported among the women patients.

Jangam et al. carried out a cross-sectional study that included 609 women diagnosed with psychiatric disorders (96 with schizophrenia) and compared them with 100 healthy women in order to determine the incidence of childhood abuse (emotional, sexual, and physical) [57]. They used the ISPCAN Child Abuse Screening Tool-Retrospective (IC-AST-R), which is a tool for screening for the frequency and severity of abuse, characteristics of the perpetrator, and type of abuse. Emotional abuse was significantly more common among those with psychiatric disorders. There was no statistically significant difference between the two groups in terms of physical and sexual abuse. There was no statistically significant difference in abuse across the diagnostic entities. Of the women with schizophrenia, 34 (about one third) reported childhood physical abuse, 26 reported emotional abuse, and 14 endorsed sexual abuse. 

Braehler and co-workers [58] explored the association between early childhood trauma and dissociative symptoms in a cohort of first-episode psychosis patients (*n* = 62), chronic psychotic patients (*n* = 43), and non-psychotic controls (*n* = 66). Childhood trauma was assessed using the Childhood Trauma Questionnaire, and dissociative symptoms were assessed by means of the Dissociative Experiences Scale. A higher severity of dissociative symptoms was positively associated with severe childhood trauma in all three groups but was particularly marked in the group of chronic patients. No statistically significant gender differences were found in any of the groups between the severity of dissociative symptoms and childhood trauma. However, an association between physical neglect and dissociation was observed in men (but not in women). 

Kelly et al. investigated the prevalence of childhood physical abuse, as evaluated by the Childhood Trauma Questionnaire (CTQ), in men and women with schizophrenia [59]. Almost half the women (41.6%) reported childhood physical abuse; the percentage was 19.6% in men. Women reporting abuse had more severe psychotic and depressive symptoms than both the men who reported abuse and the women who did not. 

Levit et al. [60] explored the association between adverse childhood experiences and positive psychotic symptoms in patients of Latino and African ancestry in the U.S. Ninety patients with schizophrenia and two hundred and forty non-psychotic controls matched for ethnicity, gender, and age were recruited. Adverse early experiences were evaluated using the Adverse Childhood Experiences (ACEs) questionnaire. In the patients, hallucinatory symptoms were positively associated with adverse childhood experiences, especially so in women. In men, ACEs predicted the presence of delusions. The authors concluded that while their findings were correlational, they supported the hypothesis that trauma exerts pathogenic effects on persons with schizophrenia. The study results also demonstrated the fact that the effects of trauma are often gender-specific. 

All authors of research in this area concur that recall bias remains a possibility when remembering incidents from childhood, especially so when a person is severely symptomatic at the time the survey is being conducted and when the purpose of the survey is self-evident [61].

#### 3.9.2. Association of Adult Trauma and Psychopathological Symptoms in Women with Schizophrenia

Yildrim et al. [62] investigated the effects of adult traumatic experiences in a sample of schizophrenia patients. Seventy women were recruited, and trauma was assessed by the Traumatic Experiences Checklist. Physical abuse was the most common traumatic event reported (81.4%) followed by emotional abuse (78.6%) and emotional neglect (55.7%). Sexual harassment was endorsed by 28.6% of the sample, and sexual abuse was endorsed by 24.3%. Hallucinations, anxiety, and affective lability were higher in women who reported a history of sexual harassment than in those who did not. Patients with exposure to adult sexual assault showed high anxiety and anger scores. 

Aakre and collaborators [63] carried out a longitudinal study of women with both schizophrenia and substance use disorders. They found that women with this comorbidity were four times more likely to meet criteria for post-traumatic stress disorder (PTSD) than were matched women with a diagnosis of major depression. Gearon and collaborators [64] investigated traumatic life events and post-traumatic stress disorder in 54 women suffering from schizophrenia and substance use disorders. Lifetime trauma history was assessed with the Traumatic Life Events Questionnaire (TLEQ), and post-traumatic stress disorders were assessed by means of the Clinician-Administered PTSD Scale. Eighty-one per cent of the sample reported past physical abuse, and 75% per cent reported re-victimization. 

### 3.10. The Role of Intimate Partner Violence

Intimate partner violence or domestic abuse are specific forms of adult trauma. Women with schizophrenia, for many reasons (unusual levels of marital and parental stress, economic hardship, limited family and social support, prevalence of substance use, psychotic symptoms, and side effects of treatment), are most often victims of domestic abuse. Afe et al. [65] carried out a cross-sectional survey in 79 women with schizophrenia attending outpatient clinics in south Nigeria. Intimate partner violence (IPV) was assessed by using the Intimate Partner Violence questionnaire, which covers three basic forms of IPV, namely physical abuse, verbal abuse, and sexual assault, all within the preceding 12 months. Seventy-three percent of the sample reported at least one form of IPV, with verbal abuse being the most frequent (71%) followed by physical abuse (40%) and sexual abuse (19%). Age, employment status, number of children, and medication compliance were all associated with frequency of IPV. Afe et al. [66] conducted another cross-sectional study with 77 women with schizophrenia, attempting to determine factors that contributed to IVP. A sociodemographic questionnaire was used to evaluate age, occupation, employment status, relationships, income, and duration of illness. Fifty-eight women (75%) reported at least one type of IPV. The most frequent type was verbal abuse (73%) followed by physical abuse (53%) and sexual assault (25%). One conclusion was that the shorter the duration of the relationship, the higher the assault prevalence. 

Suparare et al. [67] conducted a retrospective study of 304 pregnant women with severe mental illness in Australia to investigate intimate partner violence during pregnancy. Patients were divided into two groups: schizophrenia and related disorders (*n* = 125) and bipolar disorders. The two groups were then divided again according to the absence or presence of IPV. A total of 125 women with schizophrenia were recruited. Fifty-two had suffered IPV. When compared to the general population of pregnant Australian women, patients were found to be at more than triple risk for IPV. Because patients may be reluctant to divulge information about violence at home, this study suggests that IPV be routinely screened for in pregnant women with schizophrenia. 

Leslie and collaborators [68] carried out a population-based cohort study of 4470 pregnant schizophrenia women and compared them with pregnant women without schizophrenia. Risk of an emergency department visit for interpersonal violence in pregnancy or within 1 year postpartum was explored. Clinical registry data on interpersonal violence and self-reported interpersonal violence during pregnancy were recorded. Women with schizophrenia had a higher number of visits to the emergency department due to a perinatal interpersonal violence episode compared to those without schizophrenia (3.1% vs. 0.4%). The former was most likely to self-report interpersonal violence. These findings suggest that pregnancy and the postpartum period are periods of increased vulnerability for interpersonal violence for all women, but particularly so for women with schizophrenia.

**Table 2:** Summary of the main findings on the influence of childhood and adult traumatic experiences on women with schizophrenia.

Social Risk Factors	Relevant Findings	Study Design	Reference
Adverse childhood experiences	Adverse events in childhood are associated with later suicidal behavior and auditory hallucinations.	Cross-sectional	[56]
	Association with hallucinatory symptoms are more commonly found in women than in men with schizophrenia.	Cross-sectional (part of a genomic psychiatry cohort study)	[60]
	Childhood physical and emotional abuse are most commonly reported by women with schizophrenia.	Cross-sectional	[57,59]
Sexual trauma in adulthood	Positive association was found between sexual harassment and hallucinations and affective lability.	Cross-sectional	[62]
	Women are prone to suffer re-victimization.	Prospective, longitudinal	[63]
Intimate partner violence	Sexual assault and verbal and physical abuse are associated with a higher severity of psychotic symptoms.	Cross-sectional	[65]
	Verbal abuse is the most frequent type of intimate partner violence.	Cross-sectional	[66]
	Nearly half of pregnant women with schizophrenia experience intimate partner violence.	Population-based cohort study	[68]

## 4. How Do Social Factors Act Biologically and What Works to Prevent or Reduce This Effect?

Whatever the type of social determinant, its effect must necessarily be registered by the brain to produce cognitive, perceptual, and affective signals that result in disordered behavior. The most prevalent explanation of how that happens is that social exposures trigger the stress pathway, which involves the dysregulation of the hypothalamic–pituitary–adrenal (HPA) axis and the subsequent release of glucocorticoids into the blood stream. At the same time, stress influences the rate of dopamine release, which coats specific events with meaning so that reminders of the experience repeatedly trigger a stress response [69]. The dysregulation of the mesolimbic dopamine pathways and of the HPA axis results, especially with repeated exposures, in increasing sensitivity to one or more specific psychosocial stressors, which can eventually lead to a psychotic response. This may happen by introducing a delusional explanation for the stress or by inducing stimulus-free sensory perceptions or by shutting down the expression of affect [70]. The socially induced cognitive decline seen in schizophrenia may be mediated not only by dopamine dysregulation but also by the disruption of cholinergic signaling and by interference with the balance between GABAergic interneurons and glutamatergic pyramidal cells. [71]. There is also a potential mediation between environment and brain via epigenetic mechanisms, i.e., the modification of gene expression [72]. 

If the hypothesized pathways have been accurately identified, then stress reduction techniques plus appropriate pharmacological agents should, in theory, be effective in preventing or reducing the adverse effects of social risk factors. While the effectiveness of primary prevention is difficult to prove, there are studies that examine whether secondary preventive measures improve the course of illness. Solmi et al. [73] conducted an umbrella review of 83 meta-analyses of 1246 randomized controlled trials involving almost 85,000 patients. Psychosocial interventions for schizophrenia were compared against treatment as usual (e.g., mainly dopamine blockers). The included interventions intended to reduce symptom severity, improve cognition and social function, prevent relapse and treatment discontinuation, and increase quality of life were: assertive community treatment and case management, acceptance commitment therapy, adherence therapy, cognitive behavioral therapies of various kinds, cognitive remediation, family therapies, mindfulness techniques, psychoeducation, and social skills and vocational training. Of these therapies, the only one that specifically targets stress is mindfulness, but it could be argued that most of the interventions studied lead indirectly to stress reduction. The Solmi et al. review concluded that most benefits are realized when interventions are started early in the course of illness. Cognitive behavioral therapy and cognitive remediation therapy (in conjunction with pharmacotherapy and family involvement) are supported by the evidence. Individual placement and job support was the only intervention found that improved employment-related outcomes [73].

With respect to the possibility that some interventions may work better than others for the specific needs of women, the ones studied most frequently have been stress reduction techniques. This is because it is known that men’s and women’s stress circuits operate somewhat differently and respond in sex-specific ways to different kinds of stressors [74]. For instance, in the postpartum period, females present a marked adaptation of the hypothalamic–pituitary–adrenal axis characterized by the hyposecretion of cortisol, which confers an increased vulnerability to affective dysregulation. The findings of animal-based studies are promising but not convincing because results in rodents may or may not accurately reflect what happens in humans [75]. It is thought that, in humans, the processes that differentiate the sexes are complex and that they operate at multiple levels. As a result, we do not yet know what stress interventions work best to counter risk factors for psychosis in women as opposed to men.

We do know that housing conditions, both in laboratory animals and in humans, can cause stress. Interventions such as supported housing led to reduced rates of homelessness and increased housing stability in both men and women, although research has not addressed the sometimes different housing needs of men and women. For instance, women with children may need to live in specific school districts or in close proximity to other women who can share childcare responsibilities. Their neighborhoods need to be safe for themselves and their children. Both men and women have to live in circumstances that balance the need for privacy against the distress of loneliness [76]. Housing solutions need to be individualized to protect safety, encourage socialization, and increase supportive social networks. 

Parenting is a challenge, perhaps not for laboratory animals, but it is unequivocally so for individuals with schizophrenia, and it is well known that significantly more women than men with this diagnosis are parents [77]. Parenting classes and most support groups for parents are not helpful for this population; women with schizophrenia require interventions customized to their unique needs. Programs such as the Triple P Positive Parenting Program have developed specialized interventions [78] that provide education, training in coping skills, and opportunities for sharing experiences with other participants. Such programs show that the targeted population fully engage in the programs and enjoy them [79]. It is not known, however, whether the programs are effective in reducing symptoms or aid in managing the tasks of parenting. Participants in an internet-based parenting intervention have reported better parenting and coping abilities and reduced parenting stress [80]. Home-based education support and interventions have also shown good outcomes in supporting parents with complex needs [81]. These and related issues are thoroughly discussed in a book chapter by Campbell and Poon [82]. 

Anti-violence and anti-victimization programs need to be gender-specific. A Dutch study [83] found that, in adult patients with psychosis, more men reported receiving violent threats (20.7% vs. 10.5%), whereas more women reported sexual assault (13.3% vs. 3.6%). Women were more likely than men to be victimized by a partner, friend, or family member (52.9% vs. 30.6%) as opposed to a stranger (11.8% vs. 40.3%). Women were less likely than men to not disclose violence threats to care providers. On this basis, the authors of the study recommend that abuse and victimization prevention be geared towards gender. They also suggest that programs be initiated to prevent the online victimization of patients with a psychotic disorder, which is currently on the rise. Hodgins [84] recommends that programs be instituted in first-episode programs to help reduce aggressive tendencies in patients that may inadvertently elicit a violent response from others. Such programs have not been evaluated for their effectiveness. 

The victimization of persons with schizophrenia can result from provocative behavior but is more often the result of mental illness stigma. Balon [85] reviews a book edited by Dobson and Stuart [86], in which the following most promising anti-stigma practices are reported: legislative reform, advocacy, protest, contact-based education, and public education, as well as stigma self-management and peer support. Evidence of the effectiveness of anti-stigma programs remains limited, however.

Programs aimed at protecting children, boys as well as girls, from abuse and neglect exist in child protection agencies in all regions of the world. A review on the association between childhood adversity and psychosis [87] has highlighted, however, the methodological issues that exist in the literature devoted to this topic, namely selection bias, the heterogeneity of measurement instruments, the lack of control for potential confounders, and the retrospective aspect of most studies. Sample size is a particular issue for interaction studies. Only 26.4% of studies about childhood adversity were judged to be methodologically robust. It is difficult to draw causal inferences from inadequate science and, thus, to design effective prevention. This truism holds at present for many of the preventive and rehabilitative strategies used to counter the ill effects of all social risks, not only those of child adversity.

## 5. Discussion

Social risk factors negatively affect both men and women with schizophrenia, but among them are ones that impact women to a greater extent. The results of this review show that women with severe mental illness are more likely than men to self-stigmatize. Childhood trauma is reported more often by girls, which could, in part, be an openness-to-report difference rather than an occurrence rate difference. Re-victimization in adulthood (domestic violence for instance) is considerably more prevalent in women than in men. Pregnancy, when women are at their most vulnerable because they are carrying another life, is a particularly dangerous period for re-victimization.

A strength of this review is that it also examined potential preventive measures, which need to be instituted early in order to protect against development but more convincingly against severity and relapse of schizophrenia disorder. Such measures also increase the probability of functional recovery. In addition to effective psychopharmacology, the following interventions have been shown to be effective in this population in reversing stressors, however induced: There is evidence that cognitive behavioral therapy and cognitive remediation therapy, as well as individual job placement and support, reduce psychotic symptoms, improve cognitive abilities, and counter the ill effects of unemployment. The effectiveness of parenting support has not yet been sufficiently proven to reduce symptoms in women with schizophrenia, but the evidence is awaited. This is a vital area of research not only for women but also for offspring. The effectiveness of housing support, often accompanied by assertive community treatment, has been shown in several studies to be superior to treatment as usual in the context of schizophrenia [88], but the special needs of women have not been addressed in these studies. Social network interventions have been successfully used in improving HIV risk practices, reducing smoking, increasing exercise, ameliorating diet, and helping in family planning, all of which buffer the social risks encountered by women [89]; these have to be better studied, however.

Stigma is a major social risk for both men and women with psychosis, but there is good evidence that stigmatizing attitudes and behaviors can be changed when interventions take gender differences into account. Amsalem et al. [47] demonstrated that viewing a video with appropriate content featuring someone of one’s own gender can, through identification with the protagonist, change attitudes. This is also true for reducing self-stigma, which, if confronted early in the course of illness, enhances treatment-seeking and improves prognosis.

## 6. Conclusions

This review focused on women with schizophrenia, whose needs are different in many ways from those of men with the same diagnosis. Schizophrenia is negatively affected by discrimination, immigration difficulties, social isolation, urbanicity, poor socioeconomic status, inadequate housing, and childhood as well as adult trauma, but women respond more to such specific risks compared to men. Understanding male/female needs helps to design increasingly effective prevention and intervention measures for both sexes/genders. Interventions targeting family-appropriate housing, income support geared to need, social and parenting support as required, protection from abuse, violence, and gender-specific stigma have proven to be successful in reducing stress levels and alleviating the symptoms of schizophrenia. This review of the schizophrenia literature concludes that psychosocial interventions to prevent or buffer social risks need to be individualized. 

## Data Availability

The data presented in this review are available upon request from the corresponding author.
